# Adjuvant Potential of Selegiline in Attenuating Organ Dysfunction in Septic Rats with Peritonitis

**DOI:** 10.1371/journal.pone.0108455

**Published:** 2014-09-30

**Authors:** Cheng-Ming Tsao, Jhih-Gang Jhang, Shiu-Jen Chen, Shuk-Man Ka, Tao-Cheng Wu, Wen-Jinn Liaw, Hsieh-Chou Huang, Chin-Chen Wu

**Affiliations:** 1 Department of Anesthesiology, Taipei Veterans General Hospital, and National Yang-Ming University, Taipei, Taiwan, R.O.C.; 2 Department of Anesthesiology, Tri-Service General Hospital, National Defense Medical Center, Taipei, Taiwan, R.O.C.; 3 Department of Pharmacology, National Defense Medical Center, Taipei, Taiwan, R.O.C.; 4 Department of Nursing, Kang-Ning Junior College of Medical Care and Management, Taipei, Taiwan, R.O.C.; 5 Department of Physiology, National Defense Medical Center, Taipei, Taiwan, R.O.C.; 6 Graduate Institute of Aerospace and Undersea Medicine, National Defense Medical Center, Taipei, Taiwan, R.O.C.; 7 Division of Cardiology, Department of Medicine, Taipei Veterans General Hospital, Taipei, Taiwan, R.O.C.; 8 Cardiovascular Research Center, National Yang-Ming University, Taipei, Taiwan, R.O.C.; 9 Department of Anesthesiology, Tungs' Taichung MetroHarbor Hospital, Taichung, Taiwan, R.O.C.; 10 Department of Anesthesiology, Cheng-Hsin General Hospital, Taipei, Taiwan, R.O.C.; 11 Department of Pharmacology, Taipei Medical University, Taipei, Taiwan, R.O.C.; University of Leicester, United Kingdom

## Abstract

Selegiline, an anti-Parkinson drug, has antioxidant and anti-apoptotic effects. To explore the effect of selegiline on sepsis, we used a clinically relevant animal model of polymicrobial sepsis. Cecal ligation and puncture (CLP) or sham operation was performed in male rats under anesthesia. Three hours after surgery, animals were randomized to receive intravenously selegiline (3 mg/kg) or an equivalent volume of saline. The administration of CLP rats with selegiline (i) increased arterial blood pressure and vascular responsiveness to norepinephrine, (ii) reduced plasma liver and kidney dysfunction, (iii) attenuated metabolic acidosis, (iv) decreased neutrophil infiltration in liver and lung, and (v) improved survival rate (from 44% to 65%), compared to those in the CLP alone rats. The CLP-induced increases of plasma interleukin-6, organ superoxide levels, and liver inducible nitric oxide synthase and caspase-3 expressions were ameliorated by selegiline treatment. In addition, the histological changes in liver and lung were significantly attenuated in the selegiline -treated CLP group compared to those in the CLP group. The improvement of organ dysfunction and survival through reducing inflammation, oxidative stress and apoptosis in peritonitis-induced sepsis by selegiline has potential as an adjuvant agent for critical ill.

## Introduction

Despite advances in critical care medicine, sepsis continues to be a serious clinical entity with mortality rate still 30–50% for severe sepsis [1]. Numerous clinical trials of cytokine-specific therapies failed to improve survival in patients with sepsis, however recently, using pharmacological modulators to suppress apoptosis has been shown a striking efficacy in animal models of sepsis [2]–[5].

Selegiline (SEL, L-deprenyl), a monoamine oxidase-B (MAO-B) inhibitor, is a useful anti-Parkinson drug both in monotherapy and as an adjunct to levodopa therapy [6]–[8]. The MAO-B inhibitor could protect neuronal cells by its antioxidant and anti-apoptotic effects [9], [10]. The neuroprotection effects of SEL in laboratory models may be associated with the decrease of oxidative stress, stabilization of mitochondria a)nd prevention of pro-apoptotic signaling process [11], [12]. In addition to the treatment of neurodegenerative disorder, SEL reduces brain damage and enhances recovery after stroke in rats and humans [13]–[15]. Moreover, SEL increases free radical elimination and apoptosis suppression in aged liver and collapsing heart [16], [17]. SEL has been also shown to reduce vascular permeability and lung injury in a rodent hemorrhagic shock model, mostly due its anti-apoptotic action [18]. However, no studies have shown the impact of SEL at attenuating organ dysfunction and increasing survival in sepsis. In the current study, we have tested, using a rat model of cecal ligation and puncture (CLP)-induced sepsis, the hypothesis that SEL improved survival in an intra-abdominal sepsis via its antioxidant and anti-apoptotic effects.

## Materials and Methods

### Rat model of sepsis

Seventy-two male Wistar rats (280–350 g) were used in this study. All work was approved by the Committee on the Ethics of Animal Experiments of Cheng-Hsin General Hospital (Permit Number: CHGH 99-61), and the care and handling of the animals were in adherence to the National Institutes of Health Guidelines for ethical animal treatment. Rats were bred and maintained under a 12-h light/dark cycle at a controlled temperature (21±2°C) with free access to food and tap water.

### Surgical procedures

Catheter placements of left carotid artery and right jugular vein were performed for blood pressure measuring and drugs administering, respectively. The catheters were cannulated and exteriorized to the back of the neck under anesthesia of intraperitoneal sodium pentobarbital (40–50 mg/kg) and inhalational isoflurane (0.5%–1%) given via nosecone. After surgery, the cannulated animals were allowed to recover to the normal condition overnight with standardized pellet food and tap water *ad libitum*.

The intraperitoneal sepsis was induced by CLP using methods described previously [19]. Briefly, a midline laparotomy was performed under anesthesia of intravenous pentobarbital and inhalational isoflurane. The exposed cecum was ligated with a 3-0 silk ligature just distal to the ileocecal valve, punctured twice at opposite ends with an 18-gauge needle. The cecum was replaced into the abdominal cavity and the abdominal incision was closed. In addition, 0.2% lidocaine was used to infiltrate surgical wound in each rat after incision closure for post-operative analgesia. The rats in sham control were performed laparotomy and cecal exposure without any other manipulation. All animals immediately received normal saline solution (20 mL/kg subcutaneous) after operation.

### Experimental design

Animals were divided into four groups: (1) sham operation (SOP) group: 0.9% saline (3 mL/kg intravenous for 10 min) at 3 h after sham operation, (2) SOP + SEL: SEL (3 mg/kg intravenous for 10 min) at 3 h after sham operation, (3) CLP group: same regimen of saline at 3 h after CLP surgery, and (4) CLP + SEL group: same regiment of SEL at 3 h after CLP surgery. The dose of SEL 3 mg/kg used in this study was based on our preliminary data showing that it was effective on reducing the superoxide level raised by CLP surgery in liver and lung, while 1 mg/kg of SEL did not improve the survival of CLP rats. SEL (Sigma-Aldrich, St. Louis, MO, USA) was dissolved in 0.9% saline at the concentration of 1 mg/mL.

All rats enrolled in the study were kept in the small in-house animal facility of our institute to enable optimal monitoring: the overall health status was checked every 2–3 h for signs of distress. As suggested by Nemzek et al. [20], we followed a more accurate, empirically established set of guidelines that offered, although a very narrow, yet a feasible window of opportunity for induction of death. Specifically, rats were euthanized only at the end of each experiment (at 18 h after CLP or sham surgery) or upon signs of imminent death (i.e. unresponsive to external stimuli, inability to maintain upright position/tremor and prolonged/deep hypothermia and/or agonal breathing) by using an overdose of pentobarbital (100 mg/kg, i.v.). Then, some tissue specimens of liver and lung were immediately exercised to analyze superoxide levels, Western blotting and histological changes. In addition, the survival rate at 18 h in each group was analyzed.

### Measurement of hemodynamic parameters

At 0, 3, 6, 9 and 18 h after CLP or sham surgery, the carotid artery catheter was connected to a pressure transducer (P23ID, Statham, Oxnard, CA, USA) for the measurement of phasic blood pressure, mean arterial blood pressure (MAP) and heart rate, which were displayed on a polygraph recorder (MacLab/4e, AD Instruments Pty Ltd., Castle Hill, Australia). In addition, after recording of hemodynamic parameters at 0 and 18 h, animals were intravenously given one dose of noradrenaline (NE, 1 µg/kg) to examine their pressor responses. The vasopressor reactivity was analyzed by integrating the area under the pressure waveform induced by NE bolus. In order to normalize results of pressor responses to NE in all groups, we calculated the values of pressor responses to NE at time 0 (baseline) of each group as 100%.

### Quantification of organ function and injury

At 0, 9 and 18 h after CLP or sham surgery, the arterial blood samples were collected, and then immediately replaced by equal volumes of sterile saline. The total amount of blood removed was about 2.6 mL. Some blood (160 µL) was used to analyze the levels of pH, bicarbonate (HCO_3_^−^), arterial carbon dioxide tension (PaCO_2_), base excess (BE) and potassium concentration by an arterial blood gas analyzer (AVL OPTI Critical Care Analyzer, AVL Scientific Corp., Roswell, GA, USA). In addition, 80 µL of plasma was used to analyze the biochemical parameters of liver (alanine aminotransferase (ALT) and aspartate aminotransferase (AST)) and renal (blood urea nitrogen (BUN) and creatinine) functions. In addition, plasma level of lactate dehydrogenase (LDH) was to evaluate the extent of tissue breakdown. All of these biochemical parameters were analyzed by Fuji DRI-CHEM 3030 (Fuji Photo Film Co., Ltd., Tokyo, Japan).

### Measurement of plasma interleukin-6 concentrations

The plasma samples (150 µL) obtained at 0, 9 and 18 h were used. The plasma interleukin-6 (IL-6) was measured in duplicate with an enzyme-linked immunoadsorbent assay kit (R&D Systems, Inc., Minneapolis, MN, USA) according to the manufacturer's instructions.

### Measurement of superoxide production in the plasma, liver and lung

At the end of experiment (i.e. 18 h after CLP), the plasma sample was obtained and liver and lung were freshly harvested and cleared of blood and cut into pieces. The 5×5 mm of lung and liver tissues and 100 µL of plasma were transferred to scintillation plates. These scintillation plates containing Krebs' buffer with 1.25 mM lucigenin (final volume of 250 µL) were then placed into a microplate luminometer (Hidex Microplate Luminometer, Turku, Finland) for analysis in duplicate. All tissues of liver and lung were then dried in a 95°C oven for 24 h. The results were expressed as counts per sec (in each mg of liver and lung or in each 100 µL of plasma).

### Western blot analysis of tissues

Following protein isolation, equivalent amounts of protein were loaded onto a 10% (for inducible nitric oxide synthase (iNOS)) and 12% (for caspase-3) polyacrylamide gel for electrophoresis and blotting. After being blocked for 1 h at ambient temperature, the membrane was incubated overnight at 4°C with polyclonal anti-mouse iNOS antibody (BD Transduction Laboratories, Lexington, KY, USA) or polyclonal anti-rabbit cleaved caspase 3 antibody (Cell Signaling Technology, Danvers, CO, USA) at a 1: 1000 dilution. The blots were washed and incubated with horseradish-peroxidase-coupled secondary antibody (diluted 1∶1500; BD Transduction Laboratories, Lexington, KY, USA) for 1 h at ambient temperature. The blots were then stripped and reprobed with β-actin antibody (1∶3000; BD Transduction Laboratories, Lexington, KY, USA) as internal control. Immunoreactivity was visualized using an enhanced chemiluminescence reaction kit (Amersham Pharmacia Biotech, Buckinghamshire, UK) and quantified by scanning densitometer.

### Histological assessment

Specimens of liver and lung were harvested for histological analysis as previously described [19]. Briefly, the fixed tissues were dehydrated and embedded in paraffin. Each paraffin block was processed into 4-µm-thick slices that were stained with hematoxylin and eosin. This histological alteration was quantitatively analyzed as an index of the severity of neutrophil infiltration in 5 animals of each group. The index was determined by counting the numbers of neutrophil in 10 randomly selected high-power fields evaluated by a pathologist in a blinded fashion.

### Cell cultures and cell viability assay

Human aortic endothelial cells (HAECs, Cascade Biologics) were grown in Medium 200 (Cascade Biologics) supplemented with low serum growth supplement (Cascade Biologics) in an atmosphere of 95% air and 5% CO_2_ at 37°C in plastic flasks. The final concentrations of the components in Medium 200 contained 2% fetal bovine serum (Gibco–BRL), 1 µg/mL hydrocortisone, 10 ng/mL human epidermal growth factor, 3 ng/mL human fibroblast growth factor, 10 µg/mL heparin, and 1% antibiotic–antimycotic mixture (Gibco–BRL). At confluence, the cells were subcultured at a ratio 1∶3 and used at passage numbers 3–8. Cell viability was always found to be greater than 95% by using the trypan blue exclusion method or a 3-(4,5-dimethylthiazol-2- yl)-2,5-diphenyl tetrazolium bromide assay (Sigma-Aldrich).

### Measurement of reactive oxygen species production

We determined the effect of SEL on reactive oxygen species (ROS) production in HAECs by fluorometric assay using 2′,7′-dichlorofluorescein diacetate (DCFH-DA, Molecular Probes) as a probe for the presence of H_2_O_2_. HAECs were pre-treated with SEL in 24-well plates for 18 h, and subsequently combined with 25 ng/mL of lipopolysaccharide (LPS; *E. coli* serotype 0127:B8, L3127; Sigma-Aldrich) incubation for 3 or 24 h. Cells were subjected to incubation with DCFH-DA 20 µM for 45 min after the removal of SEL. Fluorescence intensity (relative fluorescence units) was measured at 485-nm excitation and 530-nm emission using a fluorescence micro-plate reader (VICTPR2 Multilabel Readers, USA). Incubation of SEL (1–100 µg/mL) lasting 24 h had no effect on the cell viability of HAECs.

### Western Blotting analysis of HAECs

Western blot analysis was conducted to determine the changes in expression of LPS-induced iNOS by SEL. Briefly, HAECs was lysed in a buffer containing 62.5 mM Tris-HCl, 2% SDS, 10% glycerol, 0.5 mM PMSF, 2 µg/mL aprotinin, 2 µg/mL pepstatin, and 2 µg/mL leupeptin. The whole-cell lysates were subjected to SDS-polyacrylamide (8%) gel electrophoresis, followed by electroblotting. Membranes were incubated with monoclonal anti-mouse iNOS antibody (1∶1000; BD Transduction Laboratories, Lexington, KY, USA), monoclonal anti-mouse β-actin antibody (1∶10000; Chemicon, Temecula, CA, USA) for overnight, and then incubated for 2 h with a secondary antibody labeled with horseradish peroxidase. Bands were visualized by chemiluminescence detection reagents (NEN Life Science Products, Boston, MA, USA). Densitometic analysis was conducted with the Image Quant (Promega) software.

### Statistical analysis

The data are presented as mean ± SEM of *n* determinations, where *n* represents the number of animals studied. The distribution of the variables was assessed with a normality test. Data with a normal distribution were analyzed by a one-way analysis of variance (ANOVA) or two-way ANOVA for repeated measures followed, where appropriate, by a Bonferroni correction test. The score for tissue infiltration of neutrophils was compared by the Mann-Whitney U test. Kaplan-Meier estimates were constructed for overall survival, which was then analyzed by the log-rank test. A *p* value of less than 0.05 was considered to be statistically significant.

### Materials

Unless otherwise stated all chemicals used were purchased from Sigma-Aldrich.

## Results

### Survival rate

**Figure 1:**
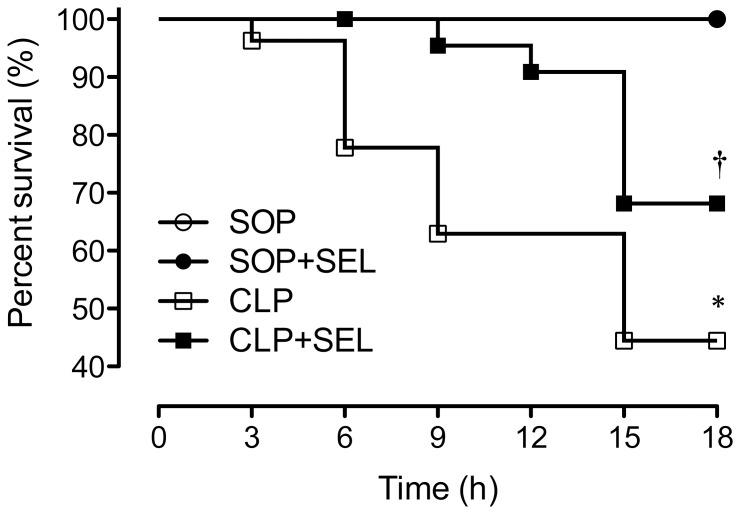
Administration of selegiline (SEL) improves survival after polymicrobial sepsis. A graph of the Kaplan-Meier survival of rats which received sham operation (SOP, n = 11), SOP and selegine administration (SOP + SEL, 3 mg/kg, i.v. at 3 h after SOP, n = 11), cecal ligation and puncture plus saline (CLP, n = 27) and CLP and SEL administration (CLP + SEL, 3 mg/kg, i.v. at 3 h after CLP, n = 23). The survival time was recorded for 18 hrs. *P<0.05, CLP vs. SOP; †*P*<0.05, with vs. without SEL in animals treated with CLP. Data are expressed as percentage of rats survived at the observed time point.

No mortality was observed within 18 h in all SOP rats (n = 11 in each SOP group, figure 1). The survival rate was significantly decreased to 63% and 44% (i.e. 17/27 and 12/27 animals) at 9 and 18 h after CLP, respectively, whereas SEL (3 mg/kg) significantly increased the survival of CLP-treated rats to 96% and 65% (i.e., 22/23 and 15/23 animals) at 9 and 18 h (*p*<0.05, vs. CLP group). However, 1 mg/kg of SEL did not improve the survival of CLP rats in our preliminary data. Because of clot or kinking of arterial catheter, blood could not be withdrawn for above tests in some of rats, esp. in rats with CLP surgery.

### Systemic hemodynamic parameters

**Figure 2:**
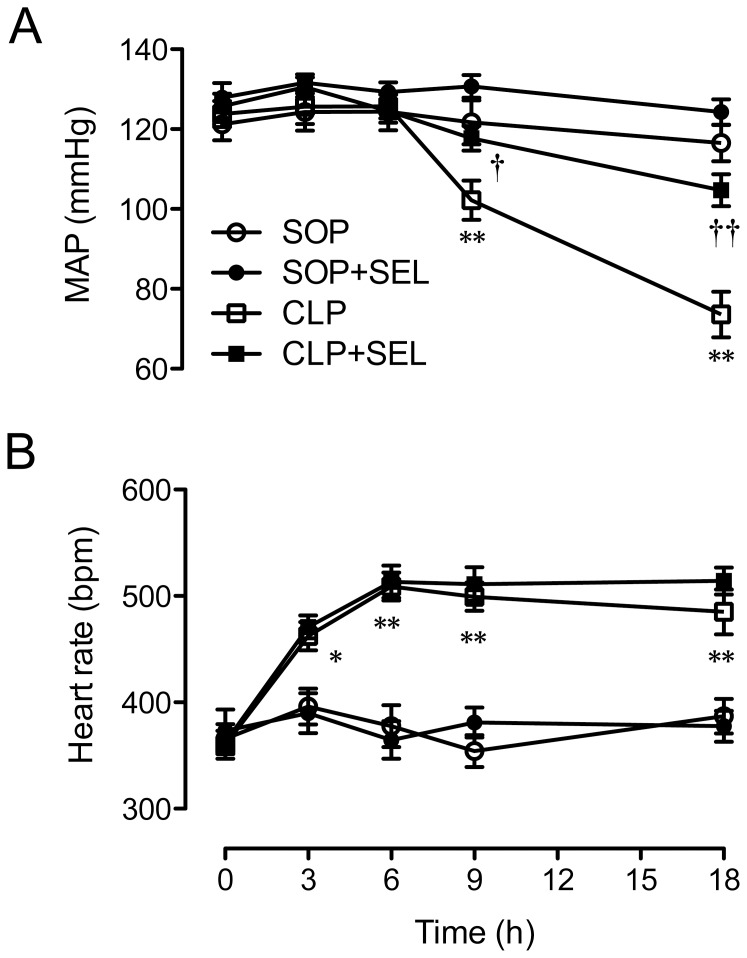
Effects of selegiline on hemodynamics. Alterations in (A) mean arterial pressure (MAP) and (B) heart rate during the experimental period. Rats underwent sham operation (SOP), SOP plus selegiline administration (3 mg/kg, i.v., SOP + SEL), cecal ligation and puncture (CLP), or CLP plus SEL administration (3 mg/kg, i.v., CLP + SEL). Data are expressed as mean ± SEM, n = 10 in each group for all time-points. *p<0.05 and **p<0.01, CLP vs. SOP; †*p*<0.05 and ††*p*<0.01, with vs. without SEL in animals treated with CLP.

As results shown in Fig. 2, the baseline values for MAP and heart rate among all groups were comparable. The CLP surgery led to a significantly substantial attenuation in MAP at 18 h (*p*<0.01, vs. SOP group; Fig. 2A), whereas significant and sustained increases in heart rate was observed from 3 h after CLP (*p*<0.05 or <0.01, vs. SOP group; Fig. 2B). The treatment with SEL significantly prevented the severe hypotension at 18 h after CLP (*p*<0.01, vs. CLP group). However, the CLP-induced tachycardia was not attenuated in rats treated with SEL. The SOP rats treated with saline or SEL exhibited stable hemodynamic conditions during the experimental period and there was no significant difference between both groups. In addition, the pressor response to NE at 18 h was significantly decreased to 19.5±2.3% of baseline in CLP rats (*p*<0.01, vs. SOP group), which was attenuated by SEL (42.9±5% of baseline; *p*<0.01, vs. CLP group). In SOP rats, the pressor response to NE at 18 h was 100.4±7.2% of baseline.

### Biochemical and blood gas parameters

Baseline values of plasma biochemical and blood gas parameters were not significantly different among all groups. The administration of SEL in the SOP groups had little effect on the biochemical and gas parameters during the experimental period.

**Figure 3:**
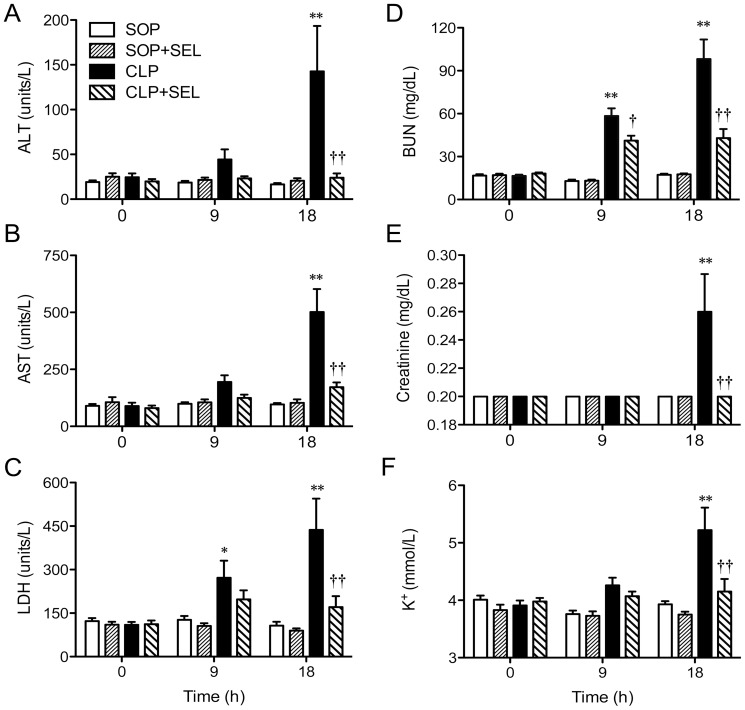
Effects of selegiline on organ function. Alterations in plasma levels of (A) alanine aminotransferase (ALT), (B) asparate aminotransferase (AST), (C) lactate dehydrogenase (LDH), (D) blood urea nitrogen (BUN), (E) creatinine, and (F) blood potassium concentration during the experimental period. Rats underwent sham operation (SOP), SOP plus selegiline administration (3 mg/kg, i.v., SOP + SEL), cecal ligation and puncture (CLP), or CLP plus SEL administration (3 mg/kg, i.v., CLP + SEL). Data are expressed as mean ± SEM, n = 10 in each group for all time-points. *p<0.05 and **p<0.01, CLP vs. SOP; †*p*<0.05 and ††*p*<0.01, with vs. without SEL in animals treated with CLP.

The CLP surgery caused time–dependent increases in plasma levels of ALT, AST, LDH, BUN, and creatinine (*p*<0.05 or 0.01, vs. SOP group; Fig. 3). These increases were significantly attenuated by the treatment of CLP rats with SEL (*p*<0.01, vs. CLP group), indicating that SEL ameliorates liver and kidney injuries induced by CLP. There was no difference in blood potassium concentration in all SOP rats. The rats treated with CLP caused hyperkalemia at 18 h (*p*<0.01, vs. SOP group; Fig. 3F). However, this hyperkalemia induced by CLP were ameliorated by SEL (*p*<0.01, vs. CLP group).

**Figure 4:**
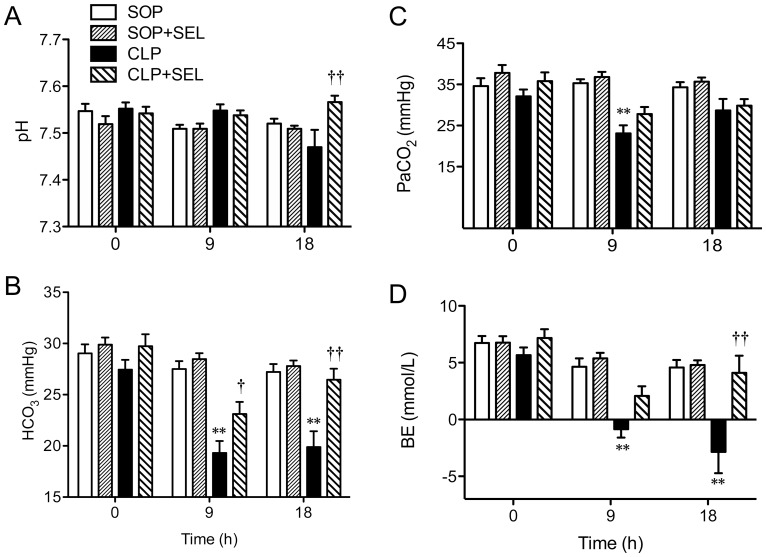
Effects of selegiline on blood potassium concentration and metabolic acidosis. Alterations in arterial blood levels of (A) pH, (B) bicarbonate (HCO_3_^−^), (C) carbon dioxide tension (PaCO_2_), and (D) base excess (BE) during the experimental period. Rats underwent sham operation (SOP), SOP plus selegiline administration (3 mg/kg, i.v., SOP + SEL), cecal ligation and puncture (CLP), or CLP plus SEL administration (3 mg/kg, i.v., CLP + SEL). Data are expressed as mean ± SEM, n = 10 in each group for all time-points. **p<0.01, CLP vs. SOP; †*p*<0.05 and ††*p*<0.01, with vs. without SEL in animals treated with CLP.

There was no difference in blood pH, HCO_3_^−^, PaCO_2_ and BE in all SOP rats. The pH level in CLP rats was decreased at 18 h, although it was not significant. However, the rats treated with CLP for 18 h caused significant decreases of HCO_3_^−^ and PaCO_2_ and decreases of BE at 9 and 18 h (*p*<0.01, vs. SOP group; Fig. 4). This indicates that CLP-induced sepsis causes compensated metabolic acidosis. However, this decrease of HCO_3_^−^ level induced by CLP was ameliorated by SEL at 9 h and 18 h (*p*<0.05 and *p*<0.01, respectively, vs. CLP group).

### Plasma IL-6 and superoxide levels in the plasma, liver, and lung

**Figure 5:**
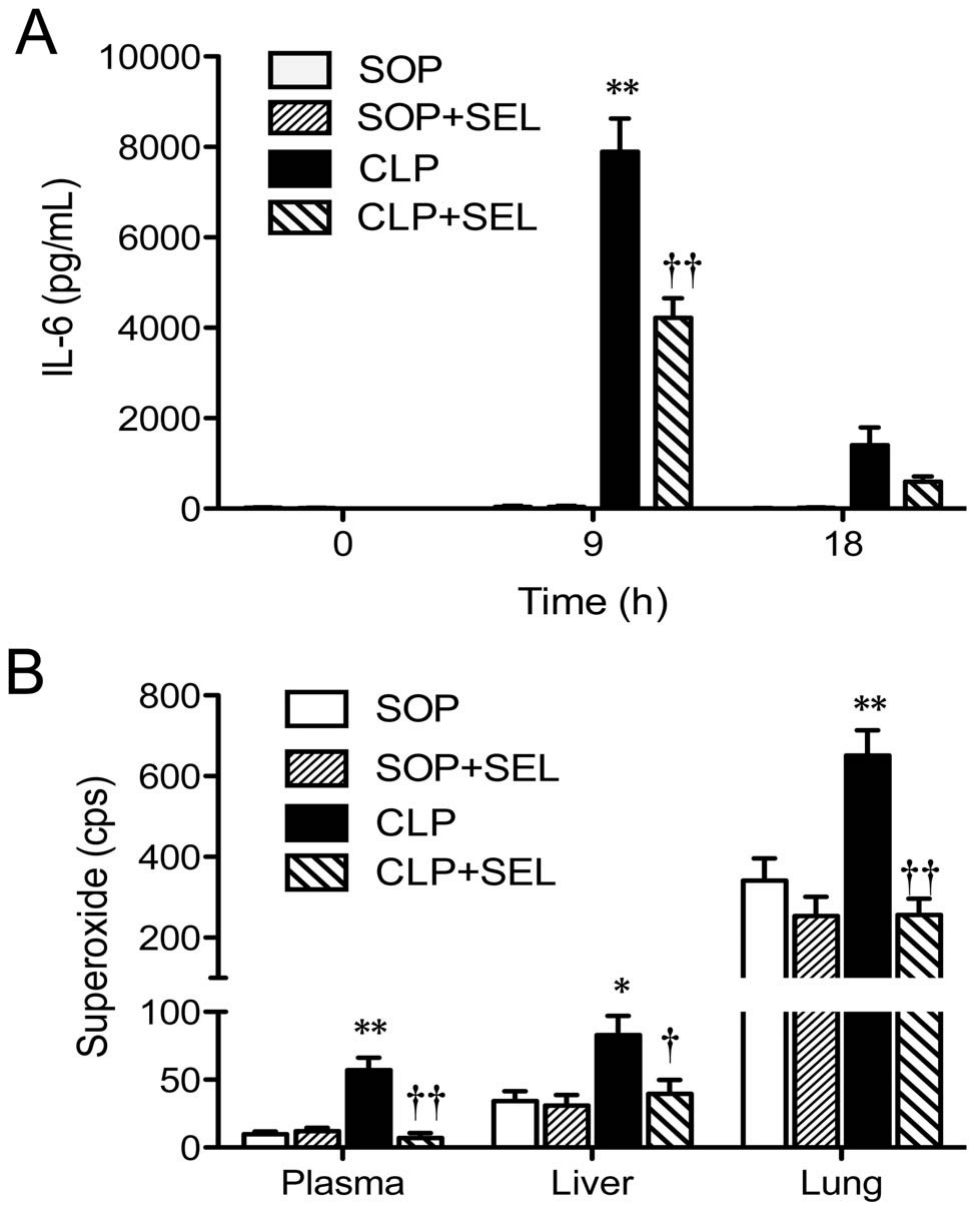
Effects of selegiline on interleukin-6 and superoxide levels. Alterations in (A) plasma interleukin-6 (IL-6) levels during the experimental period and (B) superoxide levels in plasma, liver and lung at 18 h after surgery. Rats underwent sham operation (SOP), SOP plus selegiline administration (3 mg/kg, i.v., SOP + SEL), cecal ligation and puncture (CLP), or CLP plus SEL administration (3 mg/kg, i.v., CLP + SEL). Data are expressed as mean ± SEM, n = 10 in each group for all time-points. *p<0.05 and **p<0.01, CLP vs. SOP; †*p*<0.05 and ††*p*<0.01, with vs. without SEL in animals treated with CLP.

In the SOP groups, no significant increase in plasma IL-6 level was observed during the experimental period, indicating that sham surgery and SEL treatment almost have no effect on plasma IL-6 level. The animals received CLP showed a significantly increase in the plasma level of IL-6 at 9 h (*p*<0.01, vs. SOP group; Fig. 5A), and then declined to near baseline level. However, the treatment of CLP rats with SEL significantly attenuated the increases in plasma IL-6 level at 9 h (*p*<0.01, vs. CLP group; Fig. 5A).

As results shown in Fig. 5B, animals received CLP had variable increases in the superoxide levels of plasma (*p*<0.01), liver (*p*<0.05) and lung (*p*<0.01), whereas the treatment of CLP rats with SEL significantly attenuated the production of superoxide in these specimens. However, SEL alone had no effect on the changes of these superoxide levels in the SOP group.

### iNOS and cleaved caspase-3 expression in tissues

**Figure 6:**
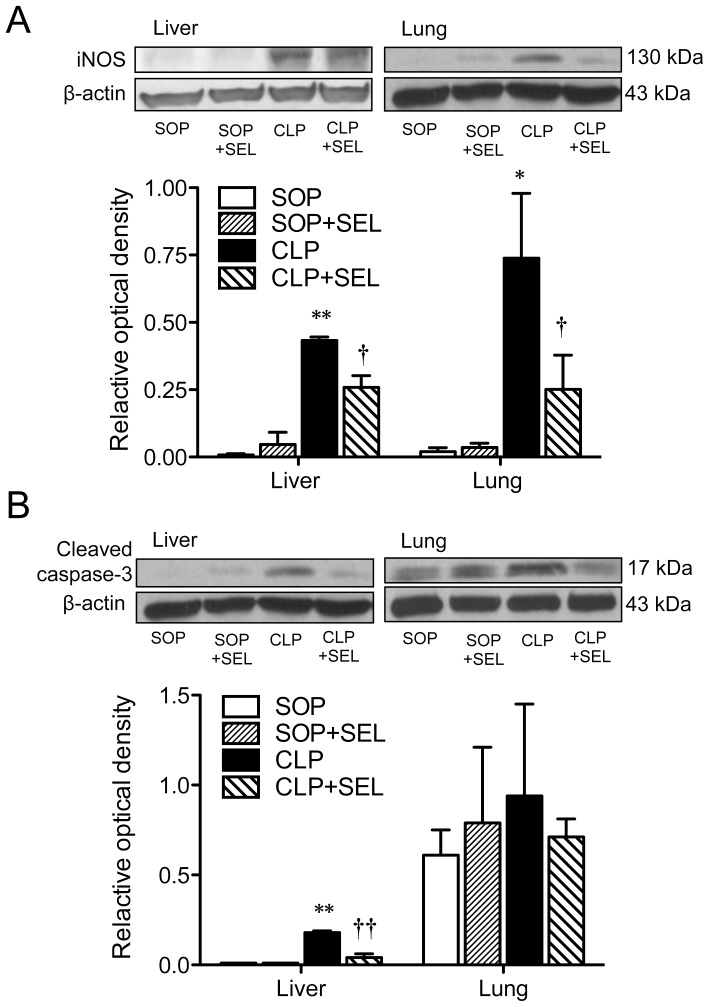
Immunoblot analysis of inducible nitric oxide synthase and cleaved caspase-3 expression in tissues. Rats underwent sham operation (SOP), SOP plus selegiline administration (3 mg/kg, i.v., SOP + SEL), cecal ligation and puncture (CLP), or CLP plus SEL administration (3 mg/kg, i.v., CLP + SEL). Liver and lung tissues were harvested at 18 h after surgery. The summary of quantification of densitometric measurement as ratio of (A) inducible nitric oxide synthase (iNOS) and (B) cleaved caspase-3 relative to β-actin is presented. Typical Western blots are shown on the upper panel of each figure. β-actin served as loading control. Data expressed as mean ± SEM, n = 3 in each group. *p<0.05 and **p<0.01, CLP vs. SOP; †*p*<0.05 and ††*p*<0.01, with vs. without SEL in animals treated with CLP.

The protein expression of iNOS was detectable in liver and lung homogenates obtained from the SOP groups, whereas a significant induction of iNOS protein was observed from the CLP rats (*p*<0.01 in the liver and *p*<0.05 in the lung, vs. SOP group; Fig. 6A). The treatment of CLP rats with SEL significantly reduced the expression of iNOS (*p*<0.05, vs. CLP group) in liver and lung. The expression of caspase-3 was significantly higher in liver from the CLP group than from the SOP group (*p*<0.01; Fig. 6B), whereas the treatment of CLP rats with SEL significantly reduced this caspase-3 expression (*p*<0.01, vs. CLP group). However, the protein expression of caspase-3 in lung homogenates was not different among all groups (Fig. 6B).

### Histopathological changes and neutrophil filtration

**Figure 7:**
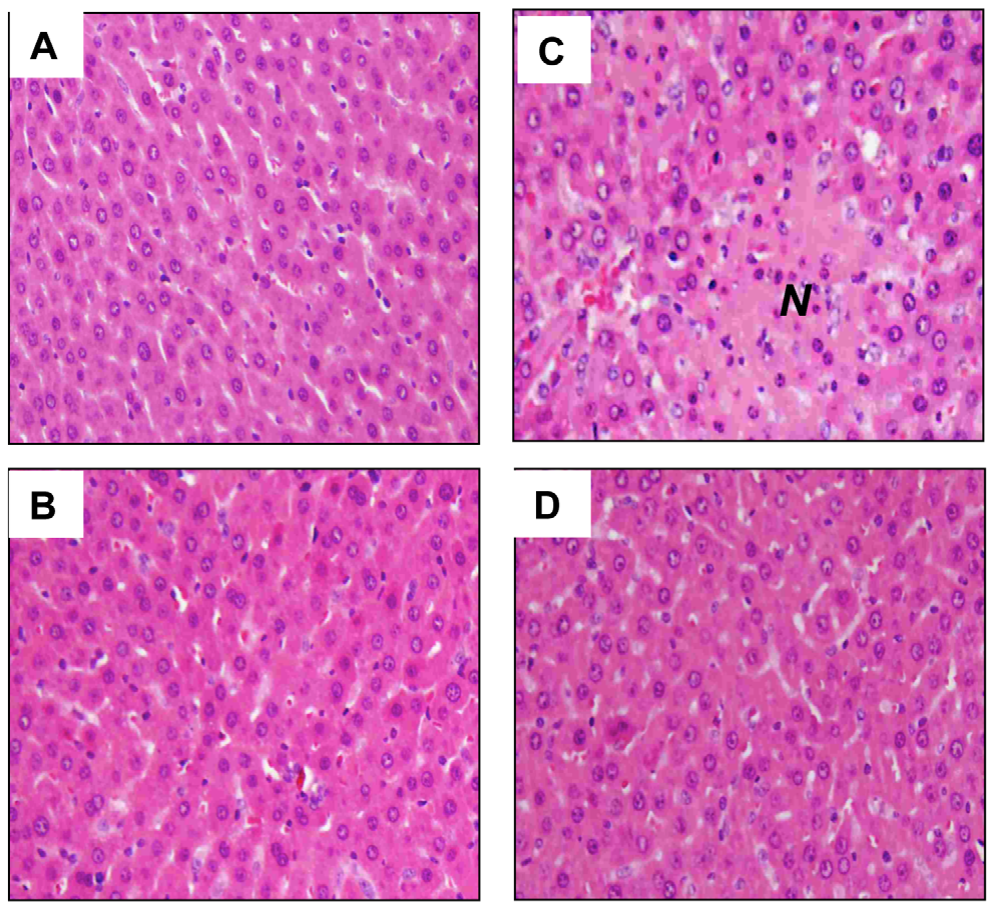
Histological analysis of liver. Liver tissue section stained with hematoxylin and eosin. Rats underwent (A) sham operation (SOP), (B) SOP plus SEL administration (3 mg/kg, i.v., SOP + SEL), (C) cecal ligation and puncture (CLP), or (D) CLP plus SEL administration (3 mg/kg, i.v., CLP + SEL). Tissues were harvested at 18 h after surgery. *N* indicates necrosis area. Shown are representative micrographs from 5 independent experiments in which the same results were obtained. Each, 400 X (original magnification).

**Figure 8:**
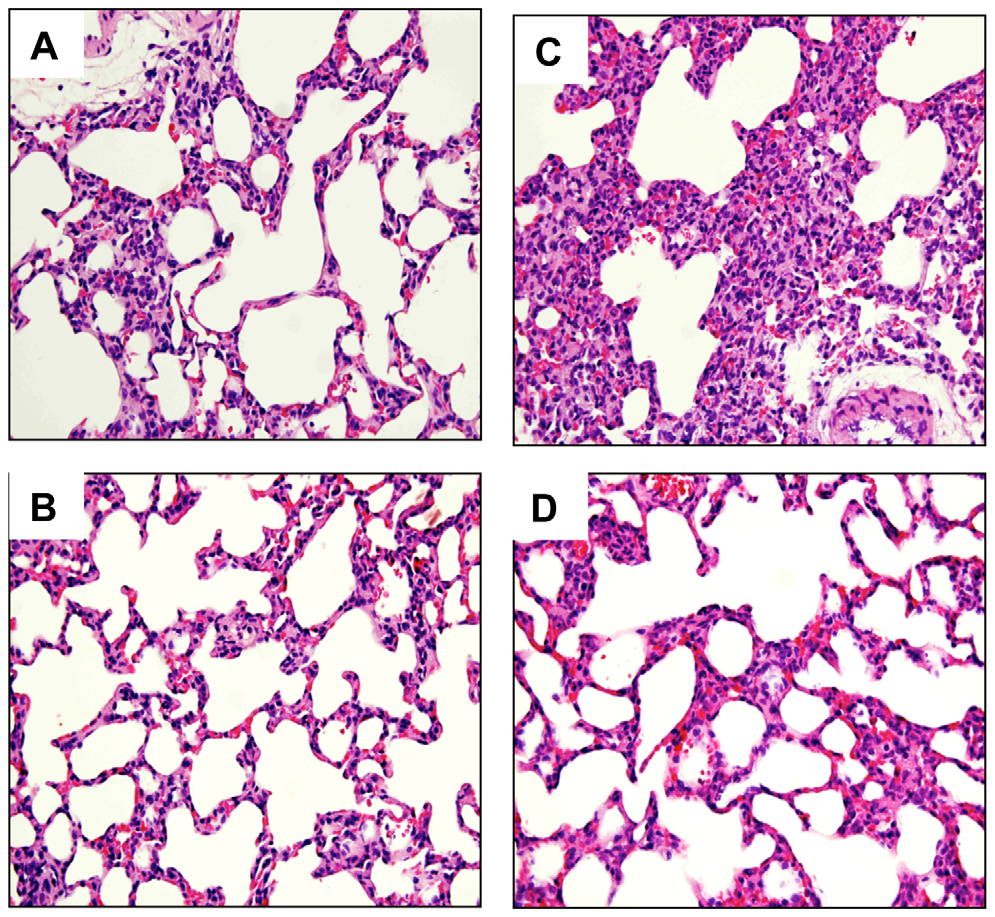
Histological analysis of lung. Lung tissue section stained with hematoxylin and eosin. Rats underwent (A) sham operation (SOP), (B) SOP plus SEL administration (3 mg/kg, i.v., SOP + SEL), (C) cecal ligation and puncture (CLP), or (D) CLP plus SEL administration (3 mg/kg, i.v., CLP + SEL). Shown are representative micrographs from 5 independent experiments in which the same results were obtained. Each, 400 X (original magnification).

Stained specimens from CLP rats revealed (i) increased interstitial edema and marked necrosis in the liver (Fig. 7), and (ii) increased interstitial edema and decreased alveolar spaces in the lung compared to those from the SOP group (Fig. 8). However, the histopathological changes in these tissues were attenuated after SEL treatment. Light microscopy only showed a little infiltration or sequestration of neutrophil in liver and lung from the SOP group, whereas overt infiltrations of neutrophil in these tissues were observed in CLP rats (2.8±0.2 in liver and 3.2±0.2 in lung; *p*<0.05). However, in CLP rats treated with SEL, the neutrophil infiltrations were significantly reduced (1.6±0.4 in liver and 2±0.3 in lung; *p*<0.05).

### ROS generation and iNOS expression in LPS-cultured HAECs

**Figure 9:**
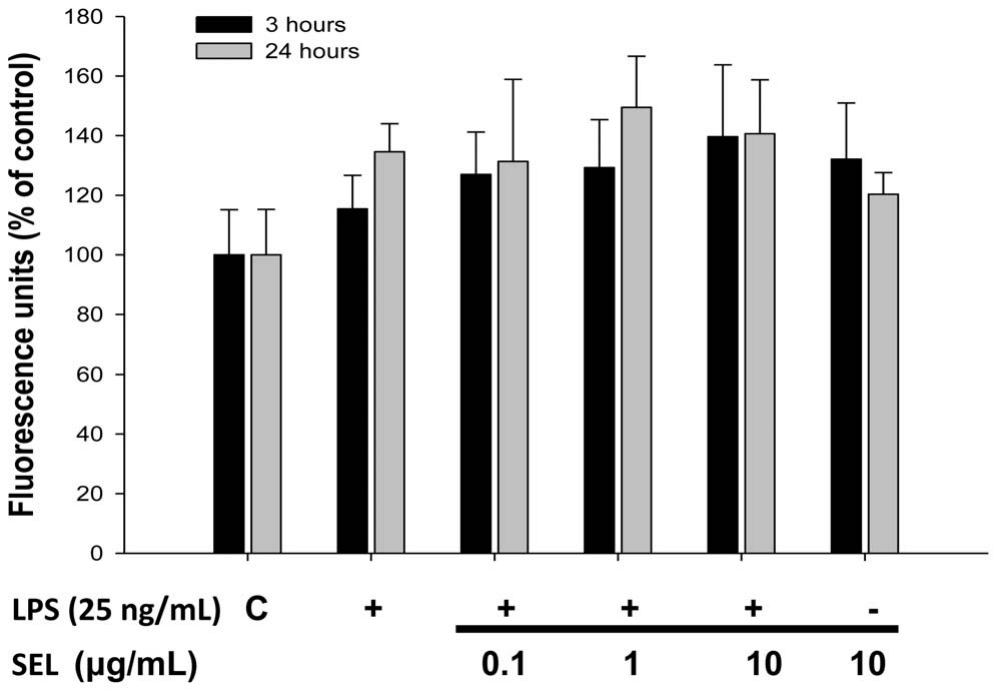
Effects of selegiline on reactive oxygen species in HAECs. Generation of reactive oxygen species induced by lipopolysaccharide (LPS) was unaffected by the treatment of selegiline (SEL) in HAECs. HAECs were pre-incubated with SEL for 18 h, followed by the incubation in 25 ng/mL of LPS for 3 or 24 h. Data are expressed as mean ± SEM of three independent experiments.

**Figure 10:**
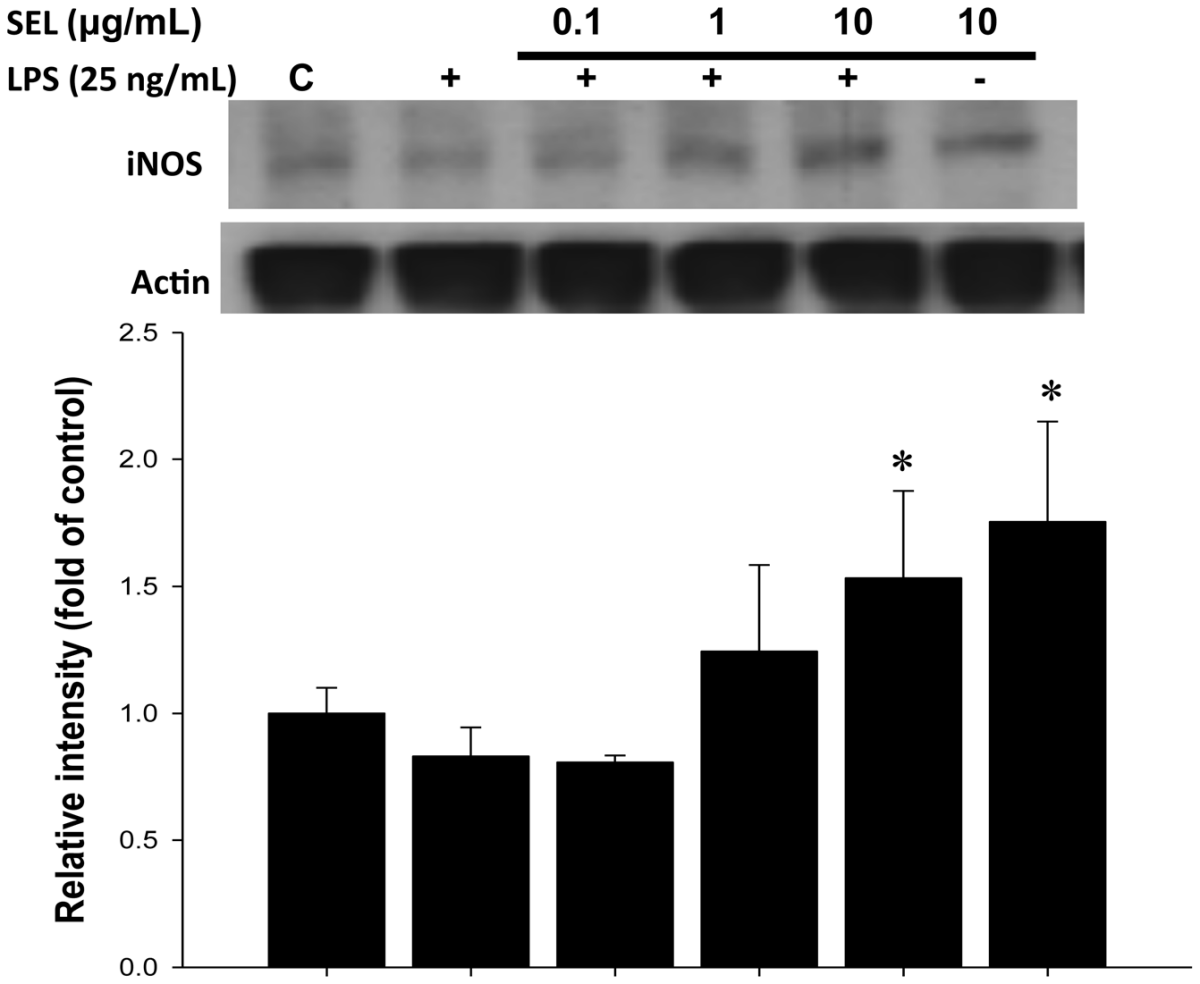
Immunoblot analysis of inducible nitric oxide synthase expression in HAECs. Selegiline (SEL) enhanced the iNOS expression in lipopolysaccharide (LPS)-cultured HAECs. HAECs were pre-treated with SEL for 18 h, and subsequently incubated in 25 ng/mL of LPS for 24 h. Data are expressed as mean ± SEM of three independent experiments. *p<0.05 compared to LPS alone.

In the present study, intracellular ROS generation was increased by LPS and was unaffected by the pretreatment of SEL (0.1–10 µg/mL) (Figure 9). Western blot analysis also showed that pretreatment of HAECs with SEL (10 µg/mL) significantly increased endothelial iNOS expression (Fig. 10) in the conditioned medium.

## Discussion

In this study, the administration of CLP-induced sepsis rats with SEL (3 mg/kg, i.v.) (i) increased arterial blood pressure and pressor response to NE, (ii) reduced plasma levels of biochemical parameters, (iii) attenuated metabolic acidosis and hyperkalemia, and (iv) ameliorated histopathological changes. This study provides novel evidence that the application of SEL seems to improve survival in the CLP-induced sepsis rats as a consequence of reduced dysfunction/injury of multiple organs. This could be due to attenuation of IL-6 production and superoxide formation and suppression of iNOS and caspase-3 expression by SEL in animals with CLP-induced sepsis.

This CLP-induced sepsis model, characterized by a biphasic process, mimics many of the pathophysiologic features of clinically relevant polymicrobial sepsis [21], [22]. An early phase results from a surge of the unbridled reactive oxygen and nitrogen species and proinflammatory cytokines mediated primarily by neutrophils, macrophages and monocytes, whereas a late phase is marked by a sustained immunosuppressive response induced primarily by apoptosis of immune, epithelial and/or endothelial cells [23]–[26].

Although multiple reactive oxygen and nitrogen species produced by neutrophils and macrophages for killing invading bacteria in the body, these species can also damage host tissues when they are produced superfluously. Furthermore, together with increased amounts of nitric oxide (NO) that are produced by the iNOS, superoxide forms the highly reactive peroxynitrite that induces irreversible damage to proteins, causing mitochondrial dysfunction and organ failure [27]. Our present study demonstrated that superoxide production and iNOS expression were increased in CLP-induced septic rats, which were attenuated by SEL administration. These observations are consistent with previous studies showing that SEL prevents the increase of oxidative products in microvascular endothelial cells exposed to burn serum [28]. However, in our *in vitro* study, pretreatment of SEL (0.1–10 µg/mL) did not attenuate intracellular ROS generation and endothelial iNOS expression in HAECs treated with LPS. Similarly, Chakravarti *et al.* also revealed that 5 nM deprenyl (i.e. SEL) in cultures of RAW 264.7 cells did not affect iNOS expression [29]. Moreover, it has been shown that pro-inflammatory cytokines can also induce excessive productions of reactive oxygen and nitrogen species [30]. Our *in vivo* data showed that CLP induced a significant increase of IL-6 in the early septic phase, which was suppressed by SEL. Thus, SEL decreased the inflammatory cytokine levels and the infiltration by neutrophils in organs (e.g. livers and lungs in this study) from sepsis animals. It has been shown that such neutrophil infiltration can lead to vascular dysfunction as well as parenchymal cell injury [31]. Based on these observations, we suggest that SEL prevents organ injury in sepsis most likely by its anti-inflammatory properties.

Indeed, we showed that SEL reduced the increased plasma levels of AST and ALT caused by CLP, which are intracellular components of liver and released into serum during ongoing cell damage. This is consistent with the histological finding showing necrotic cells in the liver from CLP rats. There is increasing evidence that, in addition to cellular necrosis, the apoptotic mode of cell death also plays a pivotal role in the pathogenesis of sepsis syndrome [24], [32]. Apoptotic cell death occurs primarily through extrinsic death-receptor pathway and/or intrinsic mitochondria pathway, which can be activated by diverse stimuli, including cytokines and free radicals [33]. The extrinsic or intrinsic pathway can activate caspase-3, leading to the degradation of cellular proteins and the destruction of cell integrity [34]. It has been shown that SEL reduces apoptosis by modulating Bcl-2 and BAX and inhibiting caspase-3 activity in a number of cell types [11], [16], [28]. Indeed, the decreased of the caspase-3 protein expression was also observed in the liver of the CLP + SEL group compared to that of the CLP group of animals in our present study. These results suggest that the beneficial effect of SEL on inflammation, tissue injury or apoptosis is further strengthened by the favorable survival outcome in the CLP group. The SEL-treated CLP animals had a 21% survival benefit over CLP controls.

In our study, SEL could attenuated caspase 3 expression in the liver of septic rats at 18 h after CLP, but not in the lung tissue. However, Tharaken et al. showed that SEL could prevent activation of caspase-3 in mesenteric vasculature in rats with hemorrhagic shock followed by 60 min of resuscitation [18]. Therefore, different experimental model, time point, and tissues may result in different effects of SEL on the caspase 3 expression.

It has been shown that amphetamine-like drugs, such as SEL and its major metabolites, L-methamphetamine and L-amphetamine, cause tachycardia [35], [36], hypotension [37], [38] or hypertension [39]. Allard *et al.* report that 3 mg/kg of SEL induces only a transient decrease in blood pressure, which returns to its baseline value after 4 min [40]. In addition, anorexia/nausea, musculoskeletal injuries, and cardiac arrhythmias occurred more often in patients receiving SEL compared with those receiving placebo [41]. Apart from these adverse effects, increased rates of elevated serum AST and ALT levels were noted [41]. However, 3 mg/kg of SEL used in this study neither affected MAP and heart rate, nor changed serum AST and ALT levels in sham control rats during the experimental period.

However, the current study has some limitations which need to be addressed. First, only one single intravenous dose of SEL was used, and consequently, we cannot exclude the possibility that multiple doses or continuous infusion could yield better outcome. Second, SEL was given at 3 h after CLP, nevertheless, the effect of SEL used in the late phase of sepsis is unknown. Third, this experimental sepsis model could not lead to profound hypoxemia at the end of study, indicating that the CLP is not a suitable experimental model of acute lung injury with significant blood gas exchange impairment, including a severe hypoxemic condition.

In conclusion, we used the most clinically relevant sepsis model to monitor sepsis-induced multiple organ dysfunction, and our findings support the hypothesis that SEL improved survival, minimized histological changes and prevented sepsis-induced multiple organ dysfunction by its anti-inflammatory and anti-apoptosis properties. This was based on the attenuation of IL-6 and superoxide production as well as the reduction of iNOS and caspase-3 expression in various tissues by SEL in animals with sepsis. Thus, we suggest that SEL could be a potential adjuvant for protecting tissues from oxidative stress and preventing organ dysfunction caused by CLP-induced sepsis.

## Supporting Information

Checklist S1 The ARRIVE Guidelines Checklist.
(PDF)
